# The association between alcohol intake and obesity in a sample of the Irish adult population, a cross-sectional study

**DOI:** 10.1186/s12889-023-16946-4

**Published:** 2023-10-24

**Authors:** Salma Rashid AlKalbani, Celine Murrin

**Affiliations:** 1 Ministry of Health, Muscat, Oman; 2 School of Public Health, Physiotherapy, and Sports Science, University College Dublin, Dublin, Ireland

**Keywords:** Obesity, Harmful alcohol drinking, Binge drinking, Waist circumference, Body Mass Index, Ireland

## Abstract

**Background:**

Obesity epidemic is one of the most serious public health challenges of the twenty-first century. Alcohol has been studied as a possible risk factor for obesity, but the evidence is discordant. This study examined the association between alcohol consumption and obesity in a sample of the Irish adult population.

**Method:**

An analytical cross-sectional study was conducted using secondary data from the 2017 Healthy Ireland Survey. The primary survey recruited patients using a two-stage probability-based technique and a face-to-face-administered questionnaire to collect data. Descriptive and comparative data were analysed to identify associations between alcohol-related variables with waist circumference (WC) and body mass index (BMI). Regression analysis was performed to examine the associations between harmful alcohol consumption (AUDIT-C score ≥ 5) (exposure variable) and obesity indicators (WC and BMI), the primary outcomes of interest. Adjustments were made for sociodemographic, health-related, and other alcohol-related variables.

**Results:**

Total of 6864 participants, aged 25 and older, took part in this survey (response rate = 60.4%). Most of the participants (81.9%) were alcohol drinkers, with the majority drinking less than three times per week (76.3%); 47.7% were considered harmful drinkers (AUDIT-C score ≥ 5). After controlling for possible confounders, positive associations of harmful alcohol consumption with WC (β = 1.72, 95% CI: 0.25, 3.19) and BMI (OR = 1.47, 95% CI: 1.10, 1.96) were observed. Binge drinking was positively associated with WC (β = 1.71, 95% CI: 0.50, 2.91), while alcohol consumption frequency was significantly and inversely associated with BMI (OR = 0.59, 95% CI: 0.44, 0.78).

**Conclusion:**

Harmful alcohol consumption was associated with obesity (high BMI, large WC) after controlling for possible confounders. Frequent binge drinkers were more likely to have a large WC, while frequent alcohol consumers were less likely to have obesity. Further longitudinal studies to examine the exact association between alcohol consumption and obesity are warranted.

## Background

Obesity has been identified as a global epidemic with significant public health implications in the twenty-first century [1]. The prevalence of obesity has more than tripled in the last four decades, with 13% of adults living with obesity and 39% of adults living with overweight [1]. It is linked to the emergence of a variety of chronic medical illnesses, including diabetes mellitus, cardiovascular diseases, and certain types of cancer [1, 2]. The prevalence of obesity varies significantly across and within nations, and this heterogeneity is driven by both individual factors and larger societal and environmental factors [3]. Because the underlying cause of obesity is multifaceted, determining the direct cause is challenging due to the interactions of various predisposing factors [4].

Alcohol intake has been examined in different epidemiological studies as a possible risk factor for the development of obesity, in addition to its link with many behavioural and mental health problems [5]. The results of studies on the association between alcohol consumption and obesity are discordant and inconclusive. While some studies have revealed a positive association [6–9] between alcohol consumption and obesity, other studies have found inverse association [10–12] or no association [13]. One large population-based study conducted in both Scotland and England examined the associations of alcohol consumption with obesity indicators (body mass index (BMI) and waist-to-hip ratio (WHR)) using a seven-day recall method. Results showed a bell-shaped curve for the association between frequency of alcohol consumption (times per month) and obesity indicators [6]. Another study among the elderly in the UK found that moderate or heavy drinkers (21 or more drinks per week) had higher adiposity levels (waist circumference (WC), WHR, BMI, and percentage-of-body-fat(%BF)) than those who consumed less than one drink per week, irrespective of the type of alcohol and pattern of consumption [9]. On the other hand, a study on French middle-aged men (50–59 years) using a self-report questionnaire found inverse associations between the frequency of alcohol consumption and obesity indicators independent of total alcohol consumed, with those who consumed alcohol occasionally (1–2 days/week) having higher odds of obesity than those who consumed alcohol more frequently (3–5 days/week) or were daily drinkers [12]. Another study looked at the effect of alcohol use on abdominal obesity in normal-weight, middle-aged (40–69 years) Korean participants, and the results showed that the frequency of alcohol consumption had no significant association with abdominal obesity in normal-weight adults [13]. The study also found that male participants who engaged in daily binge drinking (≥ 7 drinks per occasion for males, ≥ 5 drinks per occasion for females) had higher odds of abdominal obesity than less frequent binge drinkers; however, this was not observed among females [13].

Research on the association between various alcoholic beverages and obesity has shown conflicting results [9, 14–18]. A study of young and middle-aged (20–59 years) Brazilian males indicated that beer and spirit consumption were positively associated with obesity indicators (WC, WHR) but not with wine consumption [17]. Another study in France, where wine was the most common type of alcoholic beverage consumed, examined the associations between various types of alcoholic beverages and obesity (WHR, BMI). Results showed a significant association between spirit consumption and obesity [18]. However, no association was observed between beer consumption and obesity [18]. Another study among senior UK participants (60–79 years) showed that the consumption of beer, wine, spirits, and mixed drinks were all positively associated with BMI, although the greatest effect was seen with beer consumption [9]. The variability in the evidence of the link between alcohol use and obesity could be attributable to a variety of factors, including, but not limited to, the methodologies employed, the types of confounders adjusted for, the different alcohol exposure assessments (quantity, frequency, or both), the alcohol intake recall period, and the outcomes of interest (BMI, WC, WHR, %BF, or waist-to-height ratio (WHtR)) studied.

Both alcohol consumption and obesity are considered public health problems in Ireland [19]. According to the Healthy Ireland Survey 2017, 6 out of 10 adults were affected by either obesity or overweight (23% affected by obesity and 37% affected by overweight) [19]. Additionally, the proportion of individuals who consumed alcohol was 76%, of whom 39% were considered binge drinkers (≥ 6 standard units of alcohol per occasion) [19]. To the best of the author’s knowledge, no single study has examined the association between alcohol consumption and obesity in Ireland, despite their high prevalence in the country. The Alcohol Use Disorder Identification Test-Consumption (AUDIT-C) is a standardized tool used internationally to measure harmful alcohol consumption and therefore can provide more comparable and generalisable information on alcohol exposures. It is a modified version of the 10-question AUDIT instrument [20]. This study will explore the association between harmful alcohol consumption (defined as, AUDIT-C score ≥ 5) [20] and obesity in the Irish adult population using Healthy Ireland Survey 2017 data.

## Method

### Study population

The population in this study comprised those aged 25 years and older who had participated in Wave three of the Healthy Ireland Survey 2017. This survey was part of the Healthy Ireland Framework 2013–2025 that aims to improve overall wellbeing and reduce inequality within the population in Ireland [21]. Access to the anonymized, secondary data was requested from and permitted by the Irish Social Science Data Archive (ISSDA).

### Study sample and sampling strategy

The sample of the parent study was selected using a two-stage probability-based methodology with the aid of An Post Ordnance Survey Ireland geographic data [21]. The sample was determined based on electronic division clusters (each containing less than 500 addresses) to ensure coverage of a wide geographical area. Stratification by region was performed. A total of 686 clusters were selected. The addresses of those who took part in previous waves of the Healthy Ireland Survey were excluded from the third wave. Within each cluster, 20 addresses were systematically selected, with a random start address and fixed interval skips. One household member was selected randomly by interviewers to take part in this survey. The survey was conducted face-to-face by trained interviewers. Verbal consent was obtained from participants aged 18 years and older, and the parents/guardians of participants under 18 years of age provided written consent before participation in the survey. All interviews were performed at participants’ homes using computer-assisted personal interviewing (CAPI). Physical measurements (weight (kg), height (cm), and waist circumference (cm)) were measured by trained staff**.** Out of 12389 eligible addresses preselected to take part in the survey, 7487 households aged 15 and older completed the interview, for an overall response rate of 60.4%. A total of 5868 (78%) participants completed the physical measurement examination [21]. For the purpose of this study only those aged 25 and older were included in this study.

### Study design

This was an observational, analytical, cross-sectional study where both descriptive and analytical data were presented.

### Ethical approval

This study was based on secondary data from wave three of the Healthy Ireland Survey 2017. The parent survey was approved by the Research Ethics Committee of the Royal College of Physicians of Ireland (RCPI) on September 18, 2014 [19]. For the current study, a request for exemption from full ethical approval was obtained from the Research Ethics Committee of the School of Public Health, Physiotherapy, and Sport Science at University College Dublin on 25/2/2021.

### Data collection and study instrument

Out of 134 variables presented in the original Healthy Ireland Survey 2017 dataset, twenty-three variables were included in this study. These data comprised sociodemographic data, health-related data, alcohol-related data, and physical measurement data.

### Independent variables

Independent variables included alcohol-related variables, which were obtained via face-to-face interview. In this study, participants were classified into two categories: “drinkers” and “non-drinkers.” Drinkers were defined as those who responded “Yes” to the question “Have you ever consumed an alcoholic beverage in your lifetime?” while non-drinkers were defined as those who responded, “I have never had a drink” or only “I drank a few sips of an alcoholic beverage in the past.” For the alcohol frequency question, "How frequently did you consume alcohol in the last 12 months?" and for those who reported drinking alcohol, alcohol frequency was categorized as "less than three drinks per week" and "three or more drinks per week." Binge drinking was defined as the consumption of six or more standard units per occasion for both sexes. According to the response to the question “During the last 12 months, how often did you consume the equivalent of six standard drinks on one occasion?”, and among those who reported consuming alcohol, binge drinking was categorized as “less than one occasion of binge drinking per week” and “one or more occasions of binge drinking per week”.

The Alcohol Use Disorder Identification Test-Consumption, AUDIT-C, tool was used to screen for harmful alcohol consumption [20]. Three questions included in the AUDIT-C questionnaire were as follows: “How often did you have a drink containing alcohol in the past year?”, “How many drinks containing alcohol did you have on a typical day when you were drinking in the past year?”, and “How often did you have six or more drinks on one occasion in the past year?” The results of these three questions were given scores of 0–2, 3–4, or 5 or higher. A score of 5 or higher was considered harmful drinking [14]. For the regression analysis, binary variables of harmful alcohol consumption (score < 5 and score ≥ 5) were created.

### Dependent variables

The dependent variables of interest in this study were WC and BMI, which were measured by trained staff as per standard protocol. Waist circumference was considered a continuous variable and was measured in centimetres. BMI is expressed as weight (kg) divided by height squared (m^2^). BMI was further categorized into three categories: normal weight (< 25. 0 kg/m^2^), overweight (25.0–29.9 kg/m^2^) and obesity (≥ 30.0 kg/m^2^). For the multivariable logistic regression analysis, a binary variable of BMI (< 25.0 kg/m^2^ and ≥ 25.0 kg/m^2^) was created. The WC was used as a continuous variable in the multivariable linear regression analysis.

### Sociodemographic and health-related variable

Sociodemographic data (including age, sex, marital status, level of education, employment status, urban or rural residency, having a full medical card, and having a private medical card), and health-related data (including general health condition, long-term illness, smoking status, active transportation, and frequency of fruit consumption) were examined to analyse their associations with obesity indicators. For regression analysis, binary variables were created for sociodemographic and health related variables. Answers that were recorded as “don’t know” or data missing from the original dataset were treated as missing variables and thus excluded from the analysis.

### Statistical analysis

For descriptive analysis, the categorical variables were presented as numbers and percentages (n/%). Anthropometric measurements (BMI, WC) were presented as means (standard deviations, SDs) and medians (range). BMI is further presented in categories and thus expressed in numbers and percentages.

Univariate analyses were performed to examine the associations between sociodemographic data, health-related data, alcohol-related data, and obesity indicators (BMI, WC). The means of continuous variables were compared using the independent Student’s t test. The assumption of normality, homogeneity of variance, and absence of outliers were satisfied. The difference in percentages between groups was compared using the Pearson chi-square (χ^2^) test. Variables with a p value less than 0.05 in the univariate analysis were retained and included in the multivariable analysis.

Multivariable linear regression analysis was performed to analyse the association between harmful alcohol consumption (AUDIT-C score ≥ 5) and WC while controlling for possible confounders. Four different Models were constructed in this regression analysis; with confounding variables (sociodemographic and health-related variables) with significant *p* values (*p* < 0.05) were retained and included in the subsequent Model. Model 1 included all variables, while Model 2 was adjusted for sociodemographic data. Model 3 was adjusted for the statistically significant variables in Model 2, along with health-related data (general health, long-term medical illness, smoking status, active travel, and fruit consumption). The fully adjusted Model (Model 4) included the statistically significant variables in Model 3, along with alcohol-related variables (alcohol frequency and binge drinking). The results are presented as linear regression coefficients (β) and 95% confidence intervals (95% CIs). Similarly, a multivariable logistic regression analysis was performed to assess the association between harmful alcohol consumption and BMI using the different Models described earlier. The results are presented as adjusted odds ratios (ORs) and 95% CIs. The Windows-based statistical package (SPSS version 24) was used to perform the analysis. Two-tailed tests were used, and a p value of < 0. 05 was considered statistically significant.

## Results

A total of 6864 participants aged 25 and older, who took part in Wave three of the Healthy Ireland Survey, 2017, were included in this study where female participants accounted for more than half of the study population (55.8%) (Table 1). Participants aged 45 and older accounted for nearly two thirds (63.3%)of the study population. Just over half of the participants (56.3%) were married or in a civil partnership, and nearly two-thirds (60.6%) were living in urban areas. Over a third (36.5%) of the participants had a high education level. More than half of the participants (56.0%) were employed.

**Table 1:** Sociodemographic characteristics of participants according to obesity indicators {waist circumference (WC) and body mass index (BMI)} based on Healthy Ireland Survey 2017 data

Variable	Overall (*n* = 6864) n (%)	WC (cm) (*n* = 6864)		BMI (kg/m^2^) (*n* = 6864)			
		**Mean (SD**^**1**^**)(cm)**	***p* value**	** < 25.0 n (%)**	**25.0–29.9 n (%)**	** ≥ 30.0 n (%)**	***p* value**
**Age (years)**							
25–44	2517 (36.7%)	91.7 (13.72)	** < 0.001**^**a**^	880 (35.1%)	765 (30.5%)	865 (34.5%)	** < 0.001**^**b**^
45 +	4346 (63.3%)	97.0 (14.68)		903 (20.9%)	1417 (32.7%)	2007 (46.4%)	
**Sex**							
Male	3035 (44.2%)	97.7 (12.68)	** < 0.001**^**a**^	1217 (31.9%)	987 (25.8%)	1616 (42.3%)	** < 0.001**^**b**^
Female	3828 (55.8%)	89.1 (14.18)		566 (18.8%)	1195 (39.6%)	1257 (41.7%)	
**Marital status**							
Single^2^	3000 (43.7%)	92.9 (15.21)	0.272^a^	826 (27.6%)	827 (27.6%)	1341 (44.8%)	** < 0.001**^**b**^
Married^3^	3864 (56.3%)	93.3 (13.34)		957 (24.9%)	1355 (35.2%)	1532 (39.9%)	
**Residency**							
Urban	4147 (60.6%)	92.7 (14.09)	**0.004**^**a**^	1116 (27.0%)	1340 (32.4%)	1674 (40.5%)	**0.007**^**b**^
Rural	2717 (39.6%)	93.8 (14.28)		667 (24.6%)	842 (31.1%)	1199 (44.3%)	
**Educational status**							
Low/medium	4357 (63.5%)	95.0 (14.40)	** < 0.001**^**a**^	971 (22.4%)	1371 (31.6%)	1999 (46.0%)	** < 0.001**^**b**^
High	2506 (36.5%)	90.1 (13.25)		812 (32.5%)	811 (32.5%)	874 (35.0%)	
**Employment status**							
Employed	3402 (59.0%)	93.0 (14.08)	** < 0.006**^**a**^	1703 (26.1%)	2078 (31.9%)	2735 (42.0%)	0.874^b^
Unemployed^4^	2361 (41.0%)_1_	95.5 (15.64)		1783 (26.1%)	104 (32.3%)	138 (42.9%)	
**Full medical card**							
Yes	2855 (41.6%)	95.3 (15.28)	** < 0.001**^**a**^	618 (21.7%)	829 (29.1)	1400 (49.2%)	** < 0.001**^**b**^
No	4009 (58.4%)	91.7 (13.22)		1165 (29.2%)	1353 (33.9%)	1473 (36.9%)	
**Private HI**^**5**^							
Yes	3160 (46.0%)	92.3 (13.6%)	** < 0.001**^**a**^	875 (27.8%)	1084 (34.4%)	1188 (37.8%)	** < 0.001**^**b**^
No	3704 (54.0%)	93.9 (14.6%)		908 (24.6%)	1098 (29.7%)	1685 (45.7%)	

^1^ Standard deviation; ^2^ Includes single never married, separated, divorced, or widowed; ^3^ includes civil partnership; ^4^ includes unemployed looking for a job, retired, and students; ^5^ Health insurance ^a^ 2-Sample t test; ^b^ Pearson chi-square test; level of significance: *p* < 0.05

The univariate association between sociodemographic variables and obesity indicators (WC and BMI) is presented in Table 1. Most sociodemographic variables analysed showed significant associations with both WC and BMI (*p* < 0.05). Mean WC measurements were highest among those aged 45 years and older (97.0 ± 14.68 cm, *p* < 0.001), male participants (97.7 ± 12.68 cm, *p* < 0.001), participants with low/medium educational attainment (95.0 ± 14.40 cm, *p* < 0.001) and those living in rural areas (93.8 ± 14.28 cm, *p* < 0.004). Similarly, obesity (BMI ≥ 30 kg/m^2^) was more prevalent among participants aged 45 and older (46.4%, *p* < 0.001), male participants (42.3%, *p* < 0.001), participants with low/medium educational background (46.0%, *p* < 0.001).

Table 2 presents the health-related variables, including alcohol consumption, based on obesity indicators. The majority of the participants (80.6%) reported that their health was good /very good, and more than three-quarters of the study population (79.6%) were non-smokers. Alcohol consumption was highly prevalent in this study population, with 8 out of 10 participants consuming alcohol (81.9%). However, most alcohol consumers (76.3%) reported drinking less than three times per week, and approximately one-third of them (32.1%) engaged in binge drinking at least once a week. Of the 5,141 participants who completed the AUDIT-C questionnaire, nearly half (47.7%) were classified as harmful drinkers (AUDIT-C score ≥ 5).

**Table 2:** Health-related and alcohol-related characteristics of participants according to obesity indicators {waist circumference (WC) and body mass index (BMI)} based on Healthy Ireland Survey 2017 data

Variable	Overall (*n* = 6864)	WC (cm) (*n* = 6864)		BMI (kg/m^2^) (*n* = 6864)			
	**n (%)**	**Mean (SD**^**1**^**) cm**	***p* value**	** < 25.0 n (%)**	**25.0–29.9 n (%)**	** ≥ 30.0 n (%)**	***p* value**
**General health**							
Good/ very good	5530 (80.6%)	92.3 (13.48)	** < 0.001**^**a**^	1548 (28.1%)	1839 (33.4%)	2119 (38.5%)	** < 0.001**^**b**^
Fair/poor	1330 (19.4%)	97.3 (16.55)		234 (17.6%)	341 (25.7%)	753 (56.7%)	
**Long-term illness**							
Yes	2398 (34.9%)	95.9 (15.41)	** < 0.001**^**a**^	476 (19.9%)	717 (30.0%)	1195 (50.0%)	** < 0.001**^**b**^
No	4456 (64.9%)	91.7 (13.28)		1306 (29.4%)	1464 (33.0%)	1670 (37.6%)	
**Smoking status**							
Yes^2^	1402 (20.4%)	92.2 (14.23)	**0.015**^**a**^	410 (29.4%)	388 (27.8%)	598 (42.8%)	** < 0.001**^**b**^
No	5461 (79.6%)	93.4 (14.15)		1373 (25.2%)	1794 (33.0%)	2274 (41.8%)	
**Active transportation**							
Yes^3^	473 (13.5%)	91.2 (13.74)	0.574^a^	163 (34.5%)	159 (33.6%)	151 (31.9%)	**0.012**^**b**^
No	3021 (86.5%)	91.6 (13.22)		853 (28.4%)	1021 (34.0%)	1133 (37.7%)	
**Fruit consumption**							
≥ 1 time a day	4442 (64.7%)	92.0 (13.82)	** < 0.001**^**a**^	1239 (28.0%)	1433 (32.4%)	1755 (39.6%)	** < 0.001 **^**b**^
< 1 time a day	2421 (35.3%)	95.3 (14.58)		544 (22.6%)	749 (31.1%)	1117 (46.3%)	
**Alcohol consumption**							
Yes	5622 (81.9%)	93.1 (14.02)	0.627^a^	1500 (26.8%)	1840 (32.9%)	2258 (40.3%)	** < 0.001**^**b**^
No	1242 (18.1%)	93.4 (14.94)		283 (22.8%)	342 (27.6%)	615 (49.6%)	
**Alcohol frequency**							
≥ 3 times a week	862 (23.7%)	94.3 (14.34)	**0.001**^**a**^	226 (26.2%)	295 (34.3%)	340 (39.5%)	0.611^b^
< 3 times a week	2778 (76.3%)	92.2 (13.46)		767 (27.8%)	949 (34.3%)	1047 (37.9%)	
**Binge drinking**^**4**^							
≥ 1 time a week	1040 (32.1%)	96.8 (14.06)	** < 0.001**^**a**^	213 (20.3%)	371 (35.9%)	448 (43.4%)	** < 0.001 **^**b**^
< 1 time a week	2199 (67.9%)	91.8 (13.21)		641 (29.3%)	777 (35.5%)	770 (35.2%)	
**AUDIT-C**^**5**^							
Score < 5	2688 (52.3%)	91.0 (14.02)	** < 0.001**^**b**^	808 (30.1%)	821 (30.6%)	1053 (39.3%)	** < 0.001 **^**b**^
Score ≥ 5	2453 (47.7%)	94.6 (13.65)		595 (24.4%)	885 (36.3%)	957 (39.3%)	

^1^ Standard deviation; ^2^ includes yes occasionally or yes daily; ^3^ Transportation by foot or bicycle; ^4^ six or more standard units per occasion; ^5^ Alcohol Use Disorder Identification Test—Consumption; 1Valid denominator; 2; ^a^ Independent 2-sample t test; ^b^ Pearson chi-square test; level of significance *p* < 0.05

The univariate analysis of the associations between health-related variables and obesity indicators (BMI, WC) are presented in Table 2. The results clearly demonstrate significant associations between most of the health-related variables and obesity indicators. The mean WC was highest among participants with a fair/poor general health condition (97.3 ± 16.55 cm, *p* < 0.001), participants with long-term illness (95.9 ± 15.41 cm, *p* < 0.001) and non-smokers (93.4 ± 14.15 cm, *p* = 0.015). Among those with fair/poor general health, obesity (BMI ≥ 30 kg/m^2^) was reported in over half of the participants (56.7%, *p* < 0.001).

When addressing alcohol consumption, there was no significant difference in mean WC between drinkers and non-drinkers. However, those who drank more frequently (≥ 3 times per week) had a higher mean WC (94.3 ± 14.34 cm, *P* < 0.001) than those who drank less frequently, (Table 2). Participants who engaged in binge drinking one or more times per week were more likely to have a higher mean WC (96.8 ± 14.06 cm, *p* < 0.001) than those who engaged in binge drinking less frequently. Participants with harmful drinking patterns (AUDIT-C score ≥ 5) had the highest mean WC (94.6 ± 13.65 cm, *p* < 0.001) compared to participants with nonharmful drinking patterns.

Regarding BMI, the majority of alcohol consumers were living with overweight, or obesity (32.9 and 40.3%, respectively, *p* < 0.001) Table 2. No significant association between the frequency of alcohol intake and BMI was found. However, obesity was reported in slightly more than one-third of those who engaged in binge drinking one or more times per week (43.4%, *p* < 0.001). Majority of participants who scored 5 or higher on the AUDIT-C questionnaire affected with overweight and obesity (36.3%% and 39.3%%, respectively, *p* < 0.001).

Table 3 shows the multivariable linear regression analysis results, highlighting the association between harmful alcohol consumption (AUDIT-C score ≥ 5) and WC after controlling for sociodemographic variables (age, sex, marital status, level of education, employment status, urban or rural residency, having a full medical card, and having a private medical card), health-related variables (general health, long-term illness, smoking status, active transportation, and frequency of fruit consumption), and other alcohol related variables( frequency of alcohol consumption and binge drinking). After adjusting for sociodemographic and health-related variables in Model 3, harmful alcohol consumption (AUDIT-C score ≥ 5) was positively associated with WC (β = 1.93, 95% CI: 0.87, 2.98, *p* = 0.001). In the fully adjusted Model 4 and after controlling for other alcohol-related variables (alcohol frequency and binge drinking), harmful drinking continued to be significantly associated with WC (β = 1.72, 95% CI: -0.25, 3.19, *p* = 0.022). On the other hand, after controlling for sociodemographic, health-related, and other alcohol-related variables, binge drinking was found to be significantly associated with WC, with those who engaged in binge drinking once or more per week having a WC 2.14 times higher than those who engaged in binge drinking less than once per week., (β = 1.71, 95% CI 0.50, 2.91, *p* = 0.006). There was an inverse association between the frequency of alcohol consumption and mean WC, as participants with frequent alcohol consumption had a lower mean WC than those with less frequent alcohol consumption; however, the association was not statistically significant.

**Table 3:** Multivariable linear regression analysis of the association of alcohol intake, sociodemographic, and health related factors with waist circumference (cm) based on Healthy Ireland Survey 2017 data

Variable	Model 1		Model 2		Model 3		Model 4*	
	**β (95% CI)**	***p* value**	**β (95% CI)**	***p* value**	**β (95% CI)**	***p* value**	**β (95% CI)**	***p* value**
**AUDIT-C**^**1**^** score**								
Score < 5	0.00		0.00		0.00		0.00	
Score ≥ 5	2.89 (1.25, 4.53)	**0.001**	1.34 (0.47, 2.20)	**0.002**	1.93 (0.87, 2.98)	**0.001**	1.72 (0.25, 3.19)	**0.022**
**Age group (years)**								
< 45	0.00		0.00		0.00		0.00	
45 and older	4.92 (3.2, 6.22)	** < 0.001**	4.78 (3.92, 5.63)	** < 0.001**	4.06 (3.04, 5.08)	** < 0.001**	5.04 (3.92, 6.16)	** < 0.001**
**Sex**								
Female	0.00		0.00		0.00		0.00	
Male	8.12 (6.80, 9.44)	** < 0.001**	8.06 (7.21, 8.92)	** < 0.001**	8.31 (7.27, 9.36)	** < 0.001**	8.02 (6.88, 9.16)	** < 0.001**
**Marital status**								
Single^2^	0.00		0.00					
Married^3^	1.28 (0.00, 2.56)	0.050	0.82(-0.01, 1.65)	0.054				
**Educational status**								
Low/medium	0.00		0.00		0.00		0.00	
High	-1.45(-2.82, -0.08)	0.039	-2.39(-3.30, -1.48)	** < 0.001**	-1.70(-2.74, -0.65)	**0.001**	-2.43(-3.57, -1.30)	** < 0.001**
**Urban/rural residency**								
Rural	0.00		0.00		0.00		0.00	
Urban	-0.56(-1.85, 0.73)	0.394	-0.86(-1.68, -0.03)	**0.042**	-1.09(-2.11, -0.08)	**0.035**	-0.32 (1.44, 0.79)	0.569
**Employment status**								
Unemployed^4^	0.00		0.00					
Employed	1.87 (2.91, 0.84)	** < 0.001**	-0.02(-1.93, 1.89)	0.986				
**Full medical card**								
No	0.00		0.00		0.00			
Yes	1.94 (0.10, 3.79)	**0.039**	2.38 (1.40, 3.37)	** < 0.001**	1.20(-0.15, 2.54)	0.081		
**Private HI**^**5**^								
No	0.00		0.00					
Yes	0.48(-0.95, 1.90)	0.512	0.02(-0.92, 0.96)	0.964				
**General health**								
Good/ very good	0.00				0.00		0.00	
Fair/Bad	2.38(-0.29, 5.06)	0.081			3.23 (1.17, 5.28)	**0.001**	2.18 (0.55, 3.81)	**0.009**
**Past medical history**								
No	0.00				0.00			
Yes	0.94(-0.72, 2.60)	0.266			0.66(- 0.62, 1.95)	0.311		
**Smoking status**								
No	0.00				0.00		0.00	
Yes^6^	-1.77(-3.24, -0.30)	**0.018**			-2.16(-3.37, -0.94)	**0.001**	-2.47(-3.73, -1.22)	** < 0.001**
**Active transportation**								
No	0.00				0.00			
Yes^7^	0.00(-1.74, 1.74)	0.999			-0.75(-2.16, 0.67)	0.301		
**Fruit consumption**								
< 1 time a day	0.00				0.00		0.00	
≥ 1 time a day	1.14(-0.14, 2.43)	0.820			1.68 (0.62, 2.74)	**0.002**	1.00(-0.12, 2.12)	0.079
**Alcohol consumption**								
< 3 times a week	0.00						0.00	
≥ 3 times a week	-1.08(-2.63, 0.47)	0.171					-0.41(-1.69,0.87)	0.533
**Binge drinking**^**8**^								
< 1 time a week	0.00						0.00	
≥ 1 time per week	1.18 (-0.21, 2.57)	0.096					1.71 (0.50, 2.91)	**0.006**

Model 1: Adjusted for all variables, R^2^ = 0.207

Model 2: Adjusted for sociodemographic variables, adjusted R^2^ = 0.158

Model 3: Adjusted for significant sociodemographic variables in Model 2 and health-related variables, adjusted R^2^ = 0.184

Model 4: Adjusted significant sociodemographic and health-related variables in Model 3 and alcohol related variables, adjusted R^2^ = 0.195

^*^ Fully adjusted model; ^1^ Alcohol Use Disorder Identification Test—Consumption; ^2^ Includes separated, divorced, widowed; ^3^ includes civil partnership; ^4^ includes unemployed looking for a job, retired, and pupil/student; ^5^ health insurance; ^6^ includes occasional and daily smoking; ^7^ transportation by foot or bicycle; ^8^ six or more standard units per occasion; Variables with *p* values < 0.05 were included in the subsequent model. Reference group β = 0.00; level of significance *p* < 0.05

Multivariable binary logistic regression analysis was performed to examine the association between harmful alcohol intake (AUDIT-C score ≥ 5) and overweight/obesity (BMI ≥ 25.0 kg/m^2^), controlling for sociodemographic, health-related, and other alcohol-related variables, Table 4. After adjusting for sociodemographic and health-related variables in Model 3, harmful alcohol consumption (AUDIT-C score ≥ 5) was associated with a 24% increase in the risk of obesity/overweight compared to nonharmful alcohol consumption (OR = 1.24, 95% CI: 1.04, 1.49, *p* = 0.018). Further controlling for other alcohol-related variables (Model 4), harmful alcohol drinking continued to be significantly associated with overweight/obesity (OR = 1.47, 95% CI: 1.10, 1.96, *p* = 0.009). When examining the frequency of alcohol consumption and after controlling for sociodemographic, health-related, and other alcohol-related variables, frequent alcohol consumption (≥ 3 times a week) was inversely associated with overweight/obesity (OR = 0.59, 95% CI: 0.44, 0.78, *p* < 0.001). No significant association was found between binge drinking and overweight/obesity in Model 4.

**Table 4:** Multivariable binary logistic regression analysis of the association of alcohol-related variables, sociodemographic variables, and health-related variables with overweight/obesity (BMI ≥ 25 kg/m^2^) based on Healthy Ireland Survey 2017 data

Variable	Model 1		Model 2		Model 3		Model 4*	
	**OR (95% CI)**	***p* value**	**OR (95% CI)**	***p* value**	**OR (95% CI)**	***p* value**	**OR (95% CI)**	***p* value**
**AUDIT-C**^**1**^** score**								
Score < 5	1		1		1		1	
Score 5 +	1.49 (1.12, 1.99)	**0.007**	1.21 (1.05,1.38)	**0.009**	1.24 (1.04,1.49)	**0.018**	1.47 (1.10, 1.96)	**0.009**
**Age class (year)**								
< 45	1		1		1		1	
45 and older	2.43 (1.89, 3.12)	** < 0.001**	1.89 (1.65, 2.16)	** < 0.001**	1.98 (1.66, 2.37)	** < 0.001**	2.52 (1.97, 3.23)	** < 0.001**
**Sex**								
Female	1		1		1		1	
Male	1.80 (1.42, 2.28)	** < 0.001**	1.90 (1.65, 2.19)	** < 0.001**	2.07 (1.72, 2.48)	** < 0.001**	1.84 (1.46, 2.32)	** < 0.001**
**Marital status**								
Single^2^	1		1		1		1	
Married^3^	1.38 (1.09, 1.74)	**0.007**	1.26 (1.10, 1.44)	**0.001**	1.33 (1.12,1.58)	**0.001**	1.41 (1.13, 1.78)	**0.003**
**Educational status**								
Low/medium	1		1		1		1	
High	0.88 (0.68, 1.13)	0.308	0.78 (0.67, 0.90)	**0.001**	0.82 (0.68, 0.97)	**0.025**	0.93 (0.73,1.18)	0.543
**Residency**								
Rural	1		1					
Urban	0.99 (0.78, 1.26)	0.948	0.96 (0.84, 1.10)	0.595				
**Employment status**								
Unemployed^4^	1		1					
Employed	1.68 (1.20, 2.34)	**0.002**	1.13 (0.83, 1.56)	0.436				
**Full medical card**								
No	1		1		1		1	
Yes	1.56 (1.09, 2.22)	**0.015**	1.28 (1.09, 1.51)	**0.002**	1.30 (1.04, 1.65)	0.029	1.47 (1.04, 2.07)	0.029
**Private HI**^**5**^								
No	1		1					
Yes	1.27 (0.98, 1.65)	0.069	0.98 (0.85, 1.14)	0.827				
**General health**								
Good/very good	1				1		1	
Fair/poor	1.79 (1.04,3.09)	**0.037**			1.51 (1.03, 2.21)	**0.034**	1.96 (1.16, 3.32)	**0.013**
**Long term illness**								
No	1				1			
Yes	1.22 (0.88, 1.69)	0.230			1.07 (0.85, 1.34)	0.583		
**Smoking status**								
No	1				1		1	
Yes^6^	0.85 (0.65, 1.12)	0.854			0.81 (0.66, 0.99)	**0.045**	0.84 (0.65, 1.09)	0.194
**Active transportation**								
No	1				1		1	
Yes^7^	0.92 (0.67, 1.26)	0.591			0.74 (0.58, 0.93)	**0.011**	0.90 (0.66, 1.23)	0.491
**Fruit consumption**								
< 1 time a day	1				1			
≥ 1 time a day	0.88 (0.69, 1.12)	0.294			0.85 (0.71, 1.03)	0.092		
**Alcohol consumption**								
< 3 times a week	1						1	
≥ 3 times a week	0.57 (0.42, 0.76)	** < 0.001**					0.59 (0.44, 0.78)	** < 0.001**
**Binge drinking**^**8**^								
< 1 time a week	1						1	
≥ 1 time a week	1.15 (0.88,1.50)	0.294					1.15 (0.88, 1.50)	0.306

Model 1: Hosmer–Lemeshow test (H.L.) = 0.409; Nagelkerke R^2^ = 0.125

Model 2: Adjusted for sociodemographic variables; H.L. = 0.0.913; Nagelkerke R^2^ = 0.-0.082

Model 3: Adjusted for significant sociodemographic variables in Model 2 and health-related variables; H.L. = 0.054; Nagelkerke R^2^ = 0.107

Model 4: Adjusted significant sociodemographic and health-related variables in Model 3 and alcohol related variables; H.L. = 0.679; Nagelkerke R^2^ = 0.120

^*^ Fully adjusted model; ^1^ Alcohol Use Disorder Identification Test—Consumption; ^2^ Includes separated, divorced, widowed; ^3^ includes civil partnership; ^4^ includes unemployed looking for a job, retired, and pupil/student; ^5^ health insurance; ^6^ includes occasional and daily smoking; ^7^ transportation by foot or bicycle ^8^ six or more standard units per occasion; Variables with *p* values < 0.05 were included in the subsequent model. Reference group OR = 1.00; level of significance *p* < 0.05

## Discussion

### Harmful alcohol consumption and obesity

The current study showed positive associations between harmful alcohol consumption (AUDIT-C score ≥ 5) and obesity indicators (both BMI and WC) after controlling for sociodemographic and health related variables. After controlling for binge drinking and alcohol consumption frequency, harmful alcohol consumption continued to be significantly associated with WC and overweight/obesity (BMI ≥ 25 kg/m^2^). Several studies on obesity looked at either the frequency [22] or quantity [9, 11, 12, 17, 18] of alcohol consumed, or both [10, 13], and revealed mixed results. No other study has employed the AUDIT-C questionnaire to investigate the association between alcohol consumption and obesity. Using the AUDIT-C can help eliminate the inconsistencies in the evaluation of the association between alcohol use and obesity.

The exact underlying mechanism through which alcohol consumption is associated with obesity is not fully understood. According to the existing literature, alcohol consumption is the second-highest source of energy, providing almost 29 kJ (7 kcal) of energy [23]. It has been found to have an additive effect on energy obtained from other non-alcoholic sources, which can lead to weight gain [24]. Some studies have shown that alcohol acts by increasing hunger [25–27], however, other studies have shown no significant association [28, 29]. Additionally, alcohol is a suppressant of fat oxidation, favours lipid storage, and acts as a precursor for fat synthesis [30, 31]. Furthermore, alcohol intake enhances cortisol secretion, which subsequently affects the distribution pattern of fat in the body [32]. The association between types of alcoholic beverages and obesity is controversial, with some studies showing a positive association [9, 15] and others showing an inverse association [9]. Certain types of alcoholic beverages, such as beer, have higher amounts of carbohydrates per unit of alcohol than others and thereby contribute to an increased risk of obesity [15]. Binge drinking has a catabolic effect on muscle tissue, leading to fat deposition in visceral organs and muscles [33], which further leads to the development of various noncommunicable diseases, including obesity and diabetes mellitus.

### Frequency of alcohol consumption and obesity

The current study showed an inverse association between frequency of alcohol consumption and overweight/obesity (BMI ≥ 25.0 kg/m^2^); with frequent alcohol consumers (≥ 3 times a week) being 49% less likely to be affected by overweight/obesity. However, no significant association was found between frequency of alcohol consumption and WC. Previous studies examined the association between alcohol frequency and obesity, and the results were discordant [6, 10, 11, 13, 22]. Some studies found inverse associations between the frequency of alcohol consumption (using a one-year recall period) and obesity indicators (BMI and WC) in both sexes [10, 11, 22]. A study in Scotland and England, on the other hand, found a bell-shaped association between the frequency of alcohol consumption (using a 7-day recall period) and obesity indicators (BMI, WHR), with no difference in risk observed between those who never drank and those who drank the most frequently [6]. Another study, which used a 24-hour recall period, on Korean adults aged 40 to 60, found no significant association between the frequency of alcohol consumption and obesity (WC) [13]. However, this lack of an association could be related to the lower prevalence of abdominal obesity in the study population, < 8% in both sexes; thus, an exact association might be difficult to obtain [13]. 

Several factors could explain the inverse association between alcohol frequency and obesity. First, alcohol intake might affect macronutrient absorption, leading to reduced energy intake [34]. Furthermore, alcohol consumption may stimulate thermogenesis by activating the ethanol oxidizing system, which can result in weight loss [34]. This effect may be balanced by the various metabolic changes caused by alcohol consumption as well as the additional energy obtained from alcohol consumption [10]. As a result, alcohol consumption frequency may not be directly associated with body weight or fat accumulation; rather, high alcohol consumption above the sex threshold may have an effect on abdominal obesity. Overall, based on existing research, encouraging alcohol use to lower the risk of obesity is not recommended, given that the precise mechanism is not fully understood.

### Binge drinking and obesity

Nearly one-third (32.1%) of the study population engaged in binge drinking. Univariate analysis revealed a significant association between binge drinking and obesity indicators (both BMI and WC). After controlling for sociodemographic, health-related, and other alcohol-related variables, the results revealed that frequent binge drinking (≥ 1 time per week) was significantly associated with a larger mean WC than less frequent binge drinking. However, no significant association was found between binge drinking and overweight/ obesity. Several studies have found a positive association between binge drinking and obesity, with individuals engaged in binge drinking being more likely to have a larger waist circumference [13] or suffer from obesity (BMI) [34]. However, a study conducted in the US using self-reported data on adults aged 18 with low income status did not find significant association, possibly due to the small sample size [22]. The reason for the positive association between binge drinking and obesity could be attributed to the presence of other impulsive behaviours, e.g., binge eating or abnormal eating patterns, which could confound the association between binge drinking and obesity [4].

### Explanation for conflicting results in the association between alcohol consumption and obesity

Several factors could have contributed to the inconsistent results of the alcohol-obesity association. First, the underlying cause of obesity is complex, with several factors contributing to its development [1]; however, not all of these characteristics have been researched or controlled for when assessing the association between alcohol consumption and obesity, which may bias the estimated association. The current study controlled for sociodemographic and health-related variables that could potentially confound the association between alcohol consumption and obesity. However, it did not investigate individual consumption patterns or the cultural dynamics that drive alcohol consumption, or the forms of legislation that could influence alcohol consumption. Second, epidemiological studies have examined the association between alcohol consumption and obesity using different instruments [9, 11, 12, 17, 18, 22], making comparisons among studies difficult as no single well-established method was used to measure alcohol consumption. The window period used to recall alcohol intake varied between studies, ranging from a 24-hour recall period [13, 18] to a 7-day recall period [6, 9], to a 12-month recall period [10]. However, a short-term recall period does not account for the usual drinking patterns of individuals, the context in which individuals drink, or the influence of different seasons on alcohol consumption. The current study used a 12-month recall period to examine the association between alcohol consumption and obesity, which is considered more accurate in measuring the usual consumption trends in individuals. Third, the baseline prevalence of both alcohol consumption and obesity in a study population can also contribute to inconsistencies in the results obtained when analysing this association. Fourth, the types of alcoholic beverages might also affect the life habits of individuals, including eating patterns and physical activity, subsequently affecting body weight. Those who consume beer tend to have worse dietary habits than those who consume other beverages [35]. A US study showed that individuals who drank beer tended to eat fewer fruits, vegetables, and grains than those who consumed wine [35] and were more likely to eat ready-made food [36]. Beer consumption was found to be positively associated with smoking, and the interaction of these factors might alter the effect of smoking on body weight [37]. A study showed that current smokers who were heavy drinkers (> 60 g/day) had a lower BMI than never smokers/former smokers. On the other hand, WC was largest among smokers with heavy lifetime alcohol consumption [37]. Last, most of the studies used a self-report questionnaire to collect data; however, self-report questionnaires are prone to several types of bias (nonresponse bias, recall bias, and social desirability bias), and the results may be affected by intentional and unintentional misinterpretations of the questions being asked. One study found that self-reported alcohol intake accounted for only 40–60% of total sales, clearly underestimating the real amount of alcohol consumed [37]. Specifically, heavy drinkers tend to underreport their alcohol intake [37]. The period for recall can also affect the accuracy of self-reported data. However, the data used in the current study were collected during face-to-face interviews that were conducted by trained staff with the aid of CAPI to minimize errors that might occur during the interview. However, a 12-month recall period can lead to recall bias, which cannot be avoided in studies with such a design.

### Study implication

As both alcohol drinking and obesity are prevalent in Ireland, this study has several implications. First, given that obesity is multifactorial in origin, existing national and international guidelines for obesity management have not addressed the possible association between alcohol consumption and obesity; rather, they have given general behavioural recommendations on alcohol consumption [38, 39]. This is partly because of the conflicting evidence regarding the association between alcohol consumption (both quantity and frequency) and obesity. More elucidation on the association between the consumption of alcohol and obesity is advocated in both national and international guidelines. Second, Alcohol consumption is a significant public health problem in Ireland, with one-third of participants engaging in binge drinking and more than half engaging in harmful alcohol consumption. Despite current legislation to control alcohol intake, the prevalence of harmful alcohol consumption is still high, partly due to the normative view of alcohol consumption in Ireland. To mitigate the burden of alcohol use, a multisectoral approach is recommended, including raising awareness of the negative psychological and physical effects, improving the capacity to recognize and treat harmful alcohol behaviours, and raising public awareness of the possible association between alcohol consumption and obesity. Furthermore, the Alcohol Act, which was introduced in Ireland in 2018, included policies that can mitigate and further denormalize alcohol consumption, such as minimum unit pricing, structural separation, health labelling on alcohol-containing products, restrictions on alcohol advertising and marketing, regulation of alcohol sponsorship, and restrictions on certain promotional activities [40]. Third, a more consistent methodology for examining the potential association between alcohol consumption and obesity is required to reduce inconsistency in the results obtained. A prospective study design is better for examining temporal sequences and reverse causation than a cross-sectional design, where such relationships cannot be revealed. The baseline prevalence of obesity and alcohol consumption in the study population is important to consider, as it may alter the overall association. The measurement of exposure should be standardised to improve generalizability and consistency of results. The current study was unique in that it assessed the relationship between alcohol use and obesity using the AUDIT-C questionnaire, which can eliminate inconsistencies in the results obtained. Personal interviews are also preferable to self-report questionnaires because they provide more accurate, complete, and high-quality data. The alcohol intake recall period should be long enough to accurately predict the pattern of alcohol consumption and account for periods where alcohol intake is high. Last, Investment in research on alcohol consumption and obesity is recommended, particularly in Ireland, where both are considered public health problems. This will provide a better understanding of the association between alcohol consumption and obesity, as well as refine future recommendations for alcohol consumption in a community with a high prevalence of obesity. It is also recommended to tailor alcohol consumption guidelines based on an individual's medical history.

### Strengths and limitations

This is the first study to examine the association between alcohol consumption and obesity in a nationally representative sample in Ireland. The overall response rate for the 2017 Healthy Ireland Survey was significantly high (above 60%), making the data more generalizable to the entire Irish population. Data collection, including anthropometric measurements, was done by professional personnel; therefore, the risks of misinterpretation or missing data were minimised to some extent [41]. The study used the AUDIT-C score in the analysis of the association between obesity and alcohol consumption, making the results more generalizable as the AUDIT-C is a standardized tool to measure harmful alcohol consumption [16]. Last, this study controlled for a variety of sociodemographic and health-related variables that could confound the association between alcohol consumption and obesity.

Nonetheless, there are some limitations to this study. The major limitation is the cross-sectional design, which precludes the inference of a causal relationship between harmful alcohol consumption and obesity. Additionally, this study was prone to recall bias and social desirability bias, which tend to be more prevalent in face-to-face interviews and in longer recall periods [42]. The study did not look at the type or quantity of alcohol consumed, nor did it account for other potential confounders like sleep patterns, mental health, total energy intake, or type of occupation, as these variables were not collected in the original survey, all of which can confound the results [43]. Furthermore, overweight and obesity were prevalent in nearly two-thirds of the study population, increasing the likelihood of finding an association between alcohol consumption and obesity. The current study did not stratify participants by sex to identify variations in the association between harmful alcohol use and obesity between the strata, as different sexes have different patterns of alcohol consumption [44]; and thus need to be considered in future research. Lastly, underreporting of the amount of alcohol intake is problematic in both self-reporting and face-to-face interviews, especially among heavy drinkers, which might cause significant bias [45].

## Conclusion

This is the first analytical, cross-sectional study to examine the association between alcohol consumption and obesity in Ireland using the Healthy Ireland Survey 2017 data. After adjustment for sociodemographic, health-related, and other alcohol-related variables, harmful alcohol consumption was found to be significantly associated with obesity indicators (WC, BMI). Frequent binge drinking was positively and significantly associated with mean WC, while alcohol consumption frequency was significantly associated with BMI. Further longitudinal studies are recommended to explore the causal association between alcohol consumption and obesity.

## Data Availability

The data that support the findings of this study are available from [The Irish Social Science, Data Archive (ISSDA)] but restrictions apply to the availability of these data, which were used under license for the current study, and so are not publicly available. Data are, however, available from the corresponding author upon reasonable request and with the permission of [The Irish Social Science, Data Archive (ISSDA)].
